# Supplementary material for: Deletion of wfs1 Impairs Oligodendrocyte Precursor Cells Dorsal Distribution and Myelination Through the wfs1-hmgcs1 Axis in Zebrafish
Source: Biology (Basel). 2026 Aug 16;15(16):1402. doi: 10.3390/biology15161402 (PMC13510151; doi:10.3390/biology15161402)
Supplement: Supplementary file 1 [file biology-15-01402-s001.zip › File S1-Transcriptome sequencing data and differentially expressed genes (DEGs) in wfs1-- zebrafish at 5 dpf/2.KEGG/wfs1KOvsWT/ALL/src/dre04672.html]

dre04672


- dre:100151365

- Up regulated genes

100151365\_1(4.45902602692177)

- dre:30762

- Up regulated genes

30762(1.51549567324805)
- dre:368878

- Up regulated genes

368878(2.50701188401484)
- dre:30763

- Up regulated genes

30763(5.58822372755117)
- dre:571282

- Up regulated genes

571282(1.90251155561911)

- dre:100006078

- Up regulated genes

100006078(2.32113452971516)

- Down regulated genes

100006078\_1(-5.72416507735977)

- dre:100006078

- Up regulated genes

100006078(2.32113452971516)

- Down regulated genes

100006078\_1(-5.72416507735977)

- dre:797491

- Down regulated genes

797491\_1(-1.09418588609297)

- dre:797491

- Down regulated genes

797491\_1(-1.09418588609297)

- dre:799354

- Up regulated genes

799354(4.08509859637481)

- Down regulated genes

799354\_1(-1.21394100025012)

- dre:30762

- Up regulated genes

30762(1.51549567324805)
- dre:368878

- Up regulated genes

368878(2.50701188401484)
- dre:30763

- Up regulated genes

30763(5.58822372755117)
- dre:571282

- Up regulated genes

571282(1.90251155561911)

- dre:100333914

- Up regulated genes

100333914(2.08181967903621)

Close
